# Fracture Behavior of Concrete under Chlorine Salt Attack Exposed to Freeze–Thaw Cycles Environment

**DOI:** 10.3390/ma16186205

**Published:** 2023-09-14

**Authors:** Wenhao Li, Shaowei Hu

**Affiliations:** 1 School of Civil Engineering, Chongqing University, Chongqing 400045, China; 2 Yellow River Laboratory, Zhengzhou University, Zhengzhou 450001, China

**Keywords:** concrete, freeze–thaw damage, sodium chloride solution, fracture behavior, durability

## Abstract

Fracture behavior is one of the key properties to study concrete cracking under sodium chloride attack exposed to the freeze–thaw cycles environment, which is frequently neglected. In this paper, 24 single edge notch beam specimens and 24 cubes were poured. The corresponding freeze–thaw cycles test in sodium chloride solution, standard cube compressive strength of concrete test, and three-point-bending tests were carried out. The research revealed that the fracture toughness, fracture energy, relative dynamic modulus of elasticity, and standard cube compressive strength were decreased by increasing freeze–thaw cycles under sodium chloride attack, and the damage degree of concrete caused by sodium chloride solution was deeper than that of pure water. In particular, there existed good linear correlation between the fracture behavior and imposed freeze–thaw damage for various solution. Accordingly, a more reliable damage model using fracture control parameters as damage factors was proposed.

## 1. Introduction

Using concrete as a building material is widely accepted for its good versatility and relatively low cost. However, a large number of micro cracks are produced due to the non-uniform shrinkage deformation of aggregate and mortar in forming stage, which creates the possibility of aggressive material invasion into itself [1]. The deterioration of concrete structures in corrosive environments is influenced by multiple parameters and conditions [2]. Practically, concrete structures in freeze–thaw cycles (FTC) environments suffer serious damage by the pressure of ice formation. Moreover, under the same FTC times, concrete specimens in sodium chloride (Na-Cl) solution could cause more than three times performance degradation than that of pure water [3,4,5], for the higher moisture content of concrete caused by NaCl solution lead to the improved pressure of ice formation on the surface of the concrete [6,7,8]. Furthermore, according to the related reports presented by China National Development and Reform Commission (National land consolidation plan 2016–2020), more than 1.5 billion mu saline alkali land and 1000 salt lakes are scattered in North China, which, mostly, is suffered more than 100 times of FTC per year. In such regions, buildings are seriously threatened. Therefore, investigating the concrete properties under chlorine salt (mainly Na-Cl) attack exposed to FTC environment has been a topic of civil engineering interest.

Accordingly, existing research on the concrete properties exposed to FTC environment have been abundant conducted [9,10,11,12]. Researchers have cognizance that the FTC environment leads to the decline in the mechanical properties of concrete, especially the relatively tensile strength. The crack resistance and the tensile strength of concrete ($f_{t}$) is closely related to the freeze–thaw damage and lead to the failure of concrete which is far below expectations strength, since crack resistance is relatively low because of its low tensile strength [13]. To summarize, the safety of the concrete structure with cracks under FTC environment is extremely important.

The fracture behavior is helpful to analyze the response of concrete structures with cracks to external loads and to estimate the actual bearing capacity of concrete structures, which is suitable for the analysis of concrete in FTC environment. Additionally, $G_{f}$ is the most useful parameter to evaluate the fracture behavior of concrete [14,15]. Load deformation and strain softening are frequently used to arrive at the fracture energies of concrete [16,17]. The electrical resistivity and ultrasonic pulse propagation velocity in the fracture area of concrete beams are measured, and the durability of concrete quality is evaluated using the measured parameters [18]. Moreover, the application of fracture behavior to the analysis of mechanical properties provides a rational basis for concrete failure analysis and leads to better understanding of the design methods of concrete structure.

Lately, considerable progress has been made in the fracture behavior of concrete without consider the affection of environmental factor. However, the fracture properties of concrete under Na-Cl attack exposed to FTC environment is rarely provided. Furthermore, the corresponding test method and calculation of fracture behavior under Na-Cl attack exposed to FTC environment require further investigation.

For the current study, basic experimental research on the performance of concrete subjected to FTC in pure water and Na-Cl solutions was conducted based on three-point-bending (TPB) test and standard cube compressive strength test. Firstly, a new test method for single edge notch beam (SENB) under Na-Cl attack exposed to FTC environment was proposed. The fracture behavior of SENB specimens in Na-Cl solution were further explored and compared with that of SENB specimens in pure water. Accordingly, the relationship of concrete fracture behavior between freeze–thaw damage in Na-Cl solution and pure water was investigated. Ultimately, a more reliable damage model using critical fracture toughness $K_{IC}^{S}$ as damage factors to evaluate the damage degree of concrete subjected to FTC in Na-Cl solutions was first proposed. The $K_{IC}^{S}$-based damage model was compared with the existed damage model using weight loss, RDME loss or $f_{cu.k}$ loss.

## 2. Experimental Methods

### 2.1. Materials and Test Specimens

In this paper, 24 SENB (single edge notch beam) specimens (250 mm × 50 mm × 50 mm) and 24 Standard Cube specimens (150 mm × 150 mm × 150 mm) were poured, which strictly followed the Norm DL/T 5332 [19]. The presupposed concrete mix proportions (by weight) and chemical composition of raw materials are listed in Table 1 and Table 2. Specifically, ordinary Portland cement was mixed with medium river sand and 10 mm grade coarse aggregate. Additionally, superplasticizer was added into the cement paste for its water-reducing function, which has been strongly recommended to enhance the freeze resisting property of concrete in the north of China, especially to improve the freeze–thaw damage resistance. As a result, superplasticizer was added according to 0.17% of the total mass of the cement paste, and the water/cement ratio was made to 0.4. Consequently, the concrete designed in this paper is representative.

**Figure 1:**
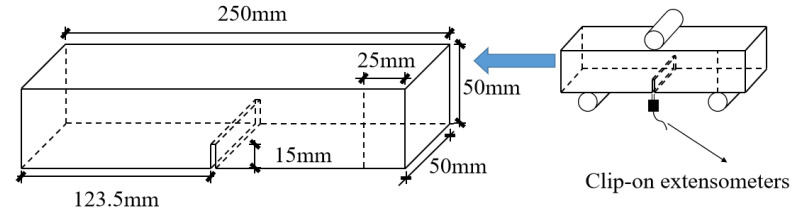
The geometry of the specimen and the test set up.

As shown in Figure 1, the pre-cut notch with 15 mm depth and 3 mm thickness was centered and perpendicular to the bottom edge of the specimen, and the crack tip opening angle was set to 60 degrees by the prefabricated steel sheet. The steel sheets were pulled out after the specimens had been poured 24 h. All of the specimens were cured in the standard-curing room for 24 days and soaked in the corresponding FTC solution for 4 days, which were introduced in Section 2.2 in detail. Additionally, 4 standard cube specimens were cast to determine $f_{cu.k}$ in 28 days. Thus, the important reference Relative dynamic modulus of elasticity (RDME) loss and compressive strength ($f_{cu.k}$) loss was obtained and then further analyzed, as seen in Section 3.3.

### 2.2. FTC Test

**Figure 2:**
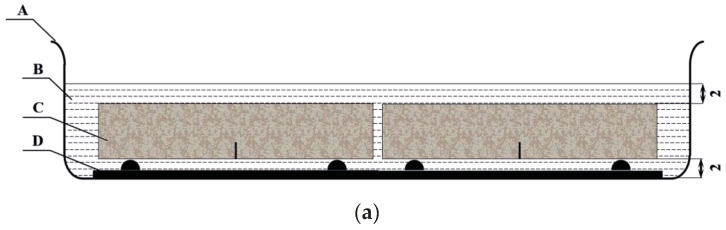

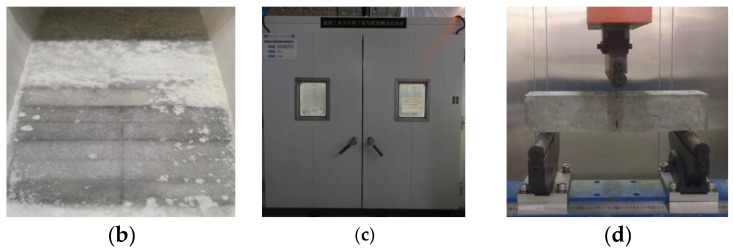
(**a**,**b**) Specimens in pool (cm): A—pool, B—solution, C—specimen, D—support; (**c**) the environmental simulation laboratory; (**d**) the TPB test.

According to the Norm GB/T 50082-2009 [20], the FTC test in Figure 2a,b showed a new test method for SENB specimens under Na-Cl attack exposed to FTC environment, which simulated the most unfavorable situation of concrete with cracks in real environment. Paraphrasing, the influence of specimen weight in FTC test was provided by the support (D part of Figure 2a) at the bottom of pool, which was located at a distance of 10% of the total length of the specimen (25 mm) from the end of the specimen; the side of the specimens with the pre-cut notch was placed downward, causing the erosion of the crack tip of the specimen to fall to the bottom of the pool, which will make the Na-Cl solution penetrate deeper into the crack tip and lead to more serious damage.

Because of the incremental freeze–thaw cycles from 0 times to 30 times, 5 groups of specimens (contain 4 SENB and cubes) were named as D, YD10, YD15, YD20, and YD30, respectively. An additional group of specimens was immersed in water with 30 FTC (SD30) to study the relationship of concrete fracture behavior between freeze–thaw damage in Na-Cl solution and pure water. A concentration of 5% Na-Cl solution was used for FTC test, as is shown in Table 3.

The pool was placed in an environmental simulation laboratory with net dimensions of 5000 mm × 4000 mm × 3000 mm, and the inside temperature could change from −40 °C to 80 °C. One cycle of the FTC test included a temperature decline from 10 ± 2 to −18 ± 2 °C with the speed of 10 ± 1 °C/h, then the temperature should be brought back to 10 ± 2 °C (and hold for one hour) with the same speed after 3 h of sustained low temperature. Moreover, a Pt (platinum thermal) sensor was embedded in the center of the specimen. According to every 5 cycles, the specimens were dried and weighed, then covered by a reconstituted solution. The maximum number of cycles was set as 30 times, which is nearly equal to the corresponding termination rules of the Norm GB/T 50082-2009 [20].

### 2.3. Testing Methodology

In the TPB test, a closed-loop servo-controlled hydraulic jack with a maximum capacity of 50 kN was employed. The clip-on extensometers were fitted at the mouth of the notch to measure the crack mouth opening displacement (CMOD), as is shown in Figure 1 and Figure 2d. To obtain the complete load–displacement (P-CMOD) curves, the testing rate was fixed at 0.025 mm/min.

## 3. Results and Discussion

### 3.1. Analysis Commonly Damage Factors

#### 3.1.1. Surface Damage

**Figure 3:**
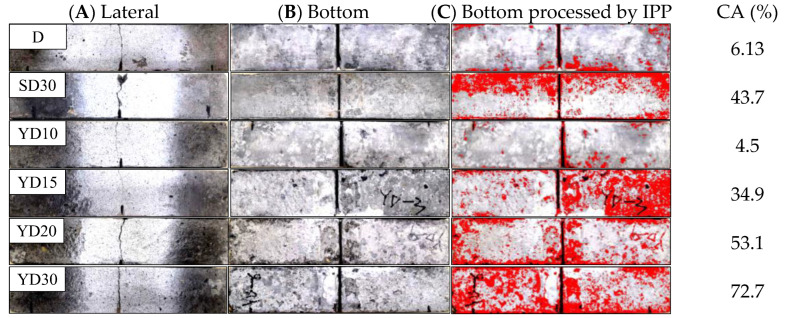
Surface deterioration of specimens.

Figure 3 shows the surface corrosion of a representative specimen in each group. The results indicated that the degree of surface deterioration was obviously deepened with the increase in the number of freeze–thaw cycles. Severe frilling of mortar and coarse aggregate was found on the surface of the SENB and cubes in the pool which was filled with Na-Cl.

Compared with group YD30, the SENB of group YD30 in pure water exhibited a distinct experimental phenomena. A slight spalling of the surface mortar was observed on the specimens of group SD30 exposed to the pure water.

Several explanations exist for the diversity of denudation degree in various FTC solution. First, the freezing of water in the large pores of concrete was expanded by FTC. It follows that the concentration difference between the unfrozen area in the large pore and the solution in the surrounding small pore were formed. Second, the water translation between the small hole and the large pore was generated by the concentration gradient. Hence, the concrete damage was primarily promoted by that translation. Third, the water content and concentration difference in the pore were exacerbated by Na-Cl, which led to more serious concrete damage. Therefore, a further study was required to obtain more reliable results.

Additionally, the coarse aggregate exposed in some severely scaled specimens immersed in the solution even peeled off. Naturally, the above results show that the Na-Cl&FTC surroundings caused serious erosion, which were even discovered universally on the coarse aggregate with high strength. An essentially deduction was put forward, that the strength difference and bond strength between mortar and aggregate is closely related to the FTC resistance of concrete. The results corroborate the findings of other researchers [11] (Lei et al. 2015). Moreover, these studies have failed to provide the fracture properties of concrete when its erosion were magnified by Na-Cl.

The calculation of corrosion area (CA) was revealed in Figure 3 to supplement the above conclusions by applying a wide-ranging software named Image Pro Plus 6.0 (IPP), which could select and calculate the pixels of the spalling area (CA) through the chromatism of the surface of the specimen. The red reach of the beam in Figure 3C were increased with numbers of freezing–thawing cycles, and the gap of CA between SD30 and YD15 were quite minor.

#### 3.1.2. Weight Loss

The following Equation (1), which was mentioned in [2], was applied to calculate the weight loss:

(1) $$\Delta W_{n}=[(W_{0}-W_{n})/W_{0}]\times 100$$

where $\Delta W_{n}$ is the weight loss of SENB at 5n FTC times (%), $W_{0}$ is the average weight of SENB before FTC (kg) and $W_{n}$ is the average weight of SENB after 5n FTC times (kg).

The weight loss of SENB submerged in Na-Cl in Table 4 exhibited three distinct stages. In Stage I, from the initial immersion to 10 FTC times, the weight increased slightly. One possible explication for this appearance may be that the porosity in SENB was amplified by FTC and that the water saturation was raised in the short duration of the Stage I. Moreover, the precipitation of Na-Cl particles on the surface of SENB had made less contribution in the weight. In Stage II, from 10 to 20 times, the weight loss increased sharply. This suggest that the accumulated surface damage under FTC suddenly emerged the denudation of SENB, which led to a more serious deterioration in Stage II. In Stage III, from 20 to 30 times, the weight loss decreased gradually. Thus, it can be concluded that the FTC damage was abated by the uniformly distribution of aggregate and mortar below the surface of SENB. In addition, the weight loss of SENB exposed in pure water showed a vastly differentiated trend, which increased gradually and smoothly.

#### 3.1.3. RDME Loss and Compressive Strength ($f_{cu.k}$) Loss

For the cube compressive test, a universal testing machine with a maximum capacity of 500 kN was employed, and the testing rate was fixed at 0.2 mm/min until failure. The dynamic modulus of elasticity (DME) was calculated by the followed Equation (2).

(2) $$E_{d}=10^{5}/(2.2+\frac{34.7}{f_{cu.k}})$$

For the specimens attacked by FTC, due to the existence of numerous micro cracks and severe surface erosion damage, it was reasonable to use Equation (2) to obtain Young’s modulus by concrete cubic compressive strength, but not the conventional test method. The RDME, which was determined by Equation (3), is the ratio of DME value to the initial DME value after 5n FTC:

(3) $$RDME=E_{dn}/E_{d0}$$

where $E_{d}$ is the DME, $E_{d0}$ is the dynamic modulus of elasticity of specimens before FTC (GPa), $E_{dn}$ is the dynamic modulus of elasticity of concrete specimens at 5n FTC (GPa).

According to the test procedure, the deterioration of the specimens was investigated by determining the RDME loss, weight loss, and compressive strength loss, and the results are shown in Table 5. The specimen was considered to be a failure if the RDME dropped to 60%, the weight loss exceeded 5%, or the compressive strength dropped to 75%.

### 3.2. Fracture Properties of Specimens Exposed to Freeze–Thaw Cycles in Salt Solution

#### 3.2.1. Calculation of Fracture Parameters

In order to prove the validation of double-K fracture model to the concrete attacked by Na-Cl in FTC circumstance. As shown in Table 6, fracture parameters were calculated by several steps.

The flexibility coefficient $c_{i}$ (µm/kN) was calculated by the average slope of the F-CMOD curve from three points, which were chosen on the initial linear segment of the F-CMOD curve before the initial cracking point.

The calculated elastic modulus E (GPa) was calculated by Equation (4).

(4) $$E=\frac{1}{tc_{i}}\left[3.70+32.60\tan^{2}(\frac{\pi }{2}\frac{a_{0}+h_{0}}{h+h_{0}})\right]$$

where t (m) is the thickness of the specimens and t = 0.05 m, $a_{0}$ (m) is the initial notch depth of the specimen and $a_{0}$ = 0.015 m, $h_{0}$ (m) is the initial crack width and $h_{0}$ = 0.003 m, h (m) is the height of the specimens and h = 0.05 m.

Then, the critical notch depth of the specimen $a_{c}$ (m) was calculated by Equation (5).

(5) $$a_{c}=\frac{2}{\pi }(h+h_{0})\arctan{\left[\frac{tEV_{c}}{32.6F_{\max}}-0.1135\right]}^{1/2}-h_{0}$$

where $V_{c}$ (µm) is the critical crack mouth opening displacement, $F_{\max}$ (kN) is the maximum load of the specimen.

The initial fracture toughness $K_{IC}^{Q}$ (MPa·m^0.5^) was calculated by Equation (6).

(6) $$\begin{array}{c} K_{IC}^{Q}=\frac{1.5(F_{Q}+\frac{mg}{2}\times 10^{-2})\times 10^{-3}\cdot S\cdot a_{0}^{1/2}}{th^{2}}f(\alpha )\\ f(\alpha )=\frac{1.99-\alpha (1-\alpha )(2.15-3.93\alpha +2.7\alpha^{2})}{(1+2\alpha ){\left(1-\alpha \right)}^{3/2}},\alpha =\frac{a_{0}}{h} \end{array}$$

where $F_{Q}$ (kN) is the initial cracking load, m (kN) is the weight of the specimen, g (m/s^2^) is the acceleration of gravity, g = 9.8 m/s^2^, S (m) is the span of the specimen, S = 0.2 m.

The critical fracture toughness $K_{IC}^{S}$ (MPa·m^0.5^) was calculated by Equation (7).

(7) $$\begin{array}{c} K_{IC}^{S}=\frac{1.5(F_{\max}+\frac{mg}{2}\times 10^{-2})\times 10^{-3}\cdot S\cdot a_{c}^{1/2}}{th^{2}}f(\alpha )\\ f(\alpha )=\frac{1.99-\alpha (1-\alpha )(2.15-3.93\alpha +2.7\alpha^{2})}{(1+2\alpha ){\left(1-\alpha \right)}^{3/2}},\alpha =\frac{a_{c}}{h} \end{array}$$

The calculation formula for fracture energy is shown in Equation (8), $G_{f}$ is fracture energy (N/mm); $W_{0}$ is each area between F and the opening displacement curve; and $A_{lig}$ is fracture ligament area—that is, the vertical projected area of the fracture surface that is parallel to the main fracture direction.

(8) $$G_{f}=w_{0}/A_{lig}$$

#### 3.2.2. Fracture Parameters Relate to FTC Times

**Figure 4:**
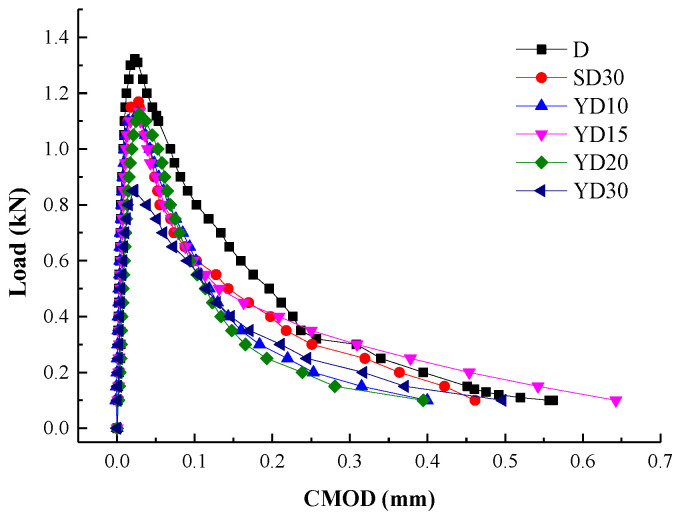
F-CMOD curves for specimens.

Fracture energy is the product of load and deflection under applied load conditions; therefore, load-deflection curves can express the fracture properties of concrete. Figure 4 shows the load deformation curves of the demonstrated SENB in each group. $F_{\max}$ of the specimen was approximately 0.87, 0.89, 0.88, 0.82, and 0.84 times than that of the D group, and $V_{C}$ of these specimens was approximately 0.79, 1.27, 1.21, 1.12, and 1.17 times than that of the D group, respectively. In particular, $F_{\max}$ and $V_{C}$ both changed significantly with increased FTC times, except $V_{C}$ decreased in specimens YD10, which possibly, for the Na-Cl particles, filled the surface pores of concrete specimens. $F_{\max}$ for SD30 group were 1.03 times more than that of YD30 group. Additionally, the slopes and area of the load-deformation curves decreased with increasing FTC times, which demonstrates that the toughness of concrete decreases with increased FTC damage because of the toughness is the area under the curve.

The structures of concrete specimens changed by the freeze–thaw cycles in salt solution; estimates showed that more microcracks are formed with ceaseless Osmotic pressure, which develop with increased FTC times. Furthermore, the independent microcracks gradually developed, and more unobstructed cracks were created. Energy was consumed with the collection and propagation of microcracks in FTC environment, which led to a decrease in fracture properties. Thus, freeze–thaw damage in Na-Cl solution significantly decreased the toughness and fracture behavior of concrete. Crack performance is well reflected in the fracture properties of concrete in Na-Cl solution.

In addition, there exists a linear correlation between fracture energy and applied FTC, as shown in Table 6, which can be used to evaluate the fracture behavior of concrete exposed to different FTC times. A slight decrease in fracture energy was observed in YD10 and SD30, and the substantial reduction was presented subsequently in YD15, YD20, YD30. This is of particular concern, as the trend of $f_{cu.k}$ loss is consistent with the trend of fracture energy.

**Figure 5:**
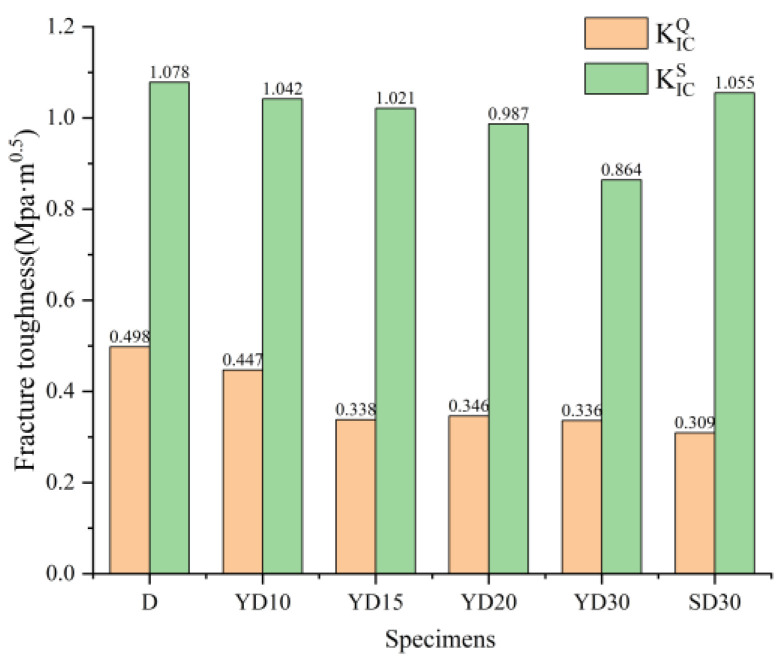
$K_{IC}^{Q}$ and $K_{IC}^{S}$ of specimens.

Figure 5 shows the $K_{IC}^{Q}$ and $K_{IC}^{S}$ of all the representative specimens of each group. No obvious trend was found between the FTC times and $K_{IC}^{Q}$. The main reason may be that the $K_{IC}^{Q}$ are insensitive with FTC. Conversely, a obviously decline was observed between $K_{IC}^{S}$ and FTC times, incrementally.

#### 3.2.3. Choosing Strain-Softening Curves

**Figure 6:**
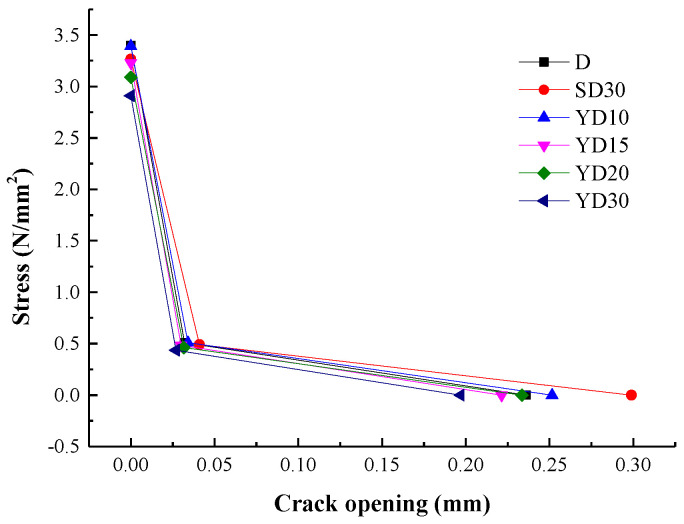
Strain-softening curves for specimens.

The characteristics of the FPZ are described by the softening constitutive curve of concrete, which is one of the important parameters to simulate the nonlinear behavior of concrete. Three indirect methods were shown and compared to obtain the appropriate softening curve. Figure 6 shows the strain-softening curves of the specimens after different FTC times, with which the main parameters ($\sigma_{s}$, $w_{s}$, $w_{0}$) were calculated using the equation presented by Petersson, CEB-FIP Model Code 1990 [21], and Reinhardt. Obviously, strain-softening curves presented by the CEB-FIP Model Code 1990 [21] and Reinhardt were highly similar, and the crack opening presented by Petersson was underestimated.

Another widely accepted method for testing the applicability of softening curves was proposed. The cohesive fracture toughness $K_{IC}^{C,e}$ and $K_{IC}^{C,t}$ were calculated by Equation (9) and Equation (10), respectively.

(9) $$K_{IC}^{C,e}=K_{IC}^{S}-K_{IC}^{Q}$$

(10) $$\begin{array}{c} K_{IC}^{C,t}=-\int_{a_{0}/a_{c}}^{1}2\sqrt{\frac{a_{c}}{\pi }}\sigma (U)F_{1}(U,V)dU,\;U=\frac{x}{a_{c}},V=\frac{a_{c}}{D}\\ F_{1}(U,V)=\frac{3.52(1-U)}{{\left(1-V\right)}^{3/2}}-\frac{4.35-5.28U}{{\left(1-V\right)}^{1/2}}+\left[\frac{1.3-0.3U^{3/2}}{{\left(1-U^{2}\right)}^{1/2}}+0.83-1.76U\right]\left[1-(1-U)V\right] \end{array}$$

where σ(U) is base on the strain-softening curves, in this paper, ${\left(K_{IC}^{C,e}-K_{IC}^{C,t}\right)}^{2}$ with different strain-softening curves were compared to test the applicability of softening curve, as shown in Table 7.

The CEB-FIP Model Code 1990 [21] was used to calculated the strain-softening curves, for this model is simpler than Reinhardt and more accurate than Petersson. Comparing the results for YD10, YD15, YD20, YD30, and SD30, it was observed that maximum applied stress and crack opening both decreased with increasing FTC times, as shown in Figure 6. For example, maximum stress and crack opening for D without applied FTC test were 0.84 and 1.17 times higher than maximum stress and crack opening after 30 FTC times (YD30); for SD30, the results were 0.82 and 1.12 times. Especially for concrete after suffering a large number of FTC times, the results became more obvious. Owing to the reduction in bite force between cement mortar and aggregate in concrete in Na-Cl&FTC, cracks more easily form and extend in concrete. Consequently, toughness and fracture behavior declined with increasing freeze–thaw damage.

#### 3.2.4. Analysis the Crack Stability of Specimens in a Salt Freeze–Thaw Environment

**Figure 7:**
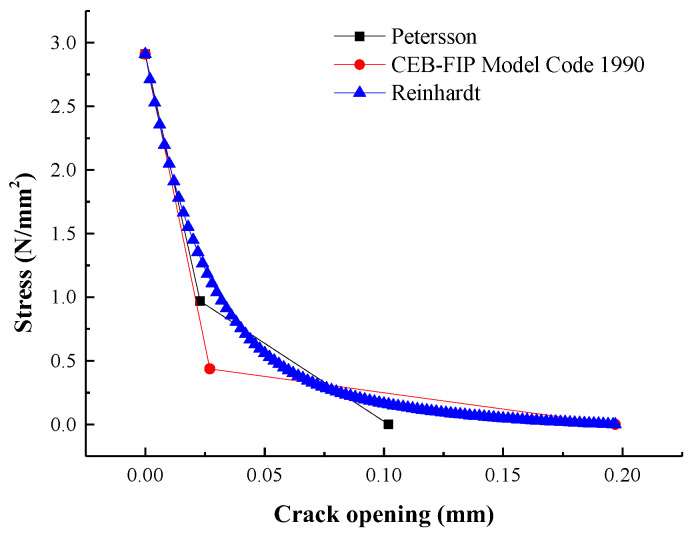
Three strain-softening curves for specimens YD30.

**Figure 8:**
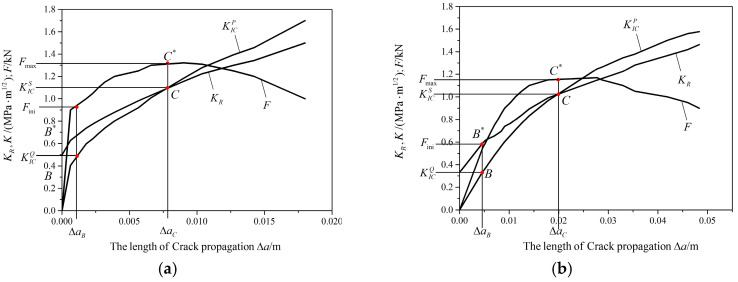

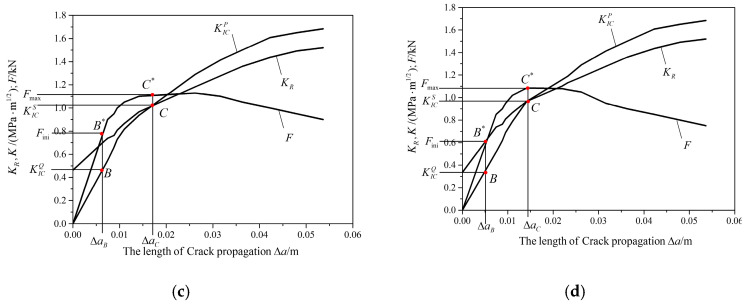
(**a**) $K_{R}$ resistance curve of D specimens; (**b**) $K_{R}$ resistance curve of SD specimens; (**c**) $K_{R}$ resistance curve of YD10 specimens; (**d**) $K_{R}$ resistance curve of YD30 specimens.

Crack stability of specimens in Na-Cl&FTC environment were studied by method of $K_{R}$ resistance curve. $K_{R}$ resistance curve can be decomposed into $K_{IC}^{Q}$ and $K_{}^{C}$, the former reflect the elastic properties of concrete, the latter was formed by the cohesive and occlusive action of aggregate in virtual equivalent fracture zone, respectively. $K_{R}(\Delta a)$ was calculated by Equation (11).

(11) $$\begin{array}{c} F_{1}(\frac{x}{a},\frac{a}{D})=\frac{3.52(1-x/a)}{{(1-a/D)}^{3/2}}-\frac{4.35-5.28x/a}{{(1-a/D)}^{1/2}}+\left[\frac{1.30-0.30{\left(x/a\right)}^{3/2}}{\sqrt{1-{\left(x/a\right)}^{2}}}+0.83-1.76\frac{x}{a}\right]\left[1-(1-\frac{x}{a})\frac{a}{D}\right]\\ \left\{\begin{array}{cc} K_{R}(\Delta a)=K_{IC}^{ini} & \left(a=a_{0}\right)\\ K_{R}(\Delta a)=K_{IC}^{ini}+\int_{a_{0}}^{a}\left[2\sigma (x)F_{1}(\frac{x}{a},\frac{a}{D})/\sqrt{\pi a}\right]dx & \left(a_{0}\leq a\leq a_{c}\right) \end{array}\right. \end{array}$$

where σ(x) was presented by CEB-FIP Model Code 1990 [21], of which the application is demonstrated in Figure 7. The stability of crack propagation was studied in Figure 8a–d. As shown in Figure 8a, $K_{R}$ curves and $K_{IC}^{P}$ curves were intersect at point C, which indicated that the state of the fracture changed from stable growth to unstable propagation after passing point C. And the corresponding point $C^{\prime }$ in F curves presented the $F_{\max}$. The same trend was found in the following graphs, which indicated that the propagation of the fracture can be described as follows:

$$\left\{\begin{array}{l} K_{R}>K_{IC}^{P}\;\mathrm{stage}\;\mathrm{of}\;\mathrm{stable}\;\mathrm{growth}\\ K_{R}=K_{IC}^{P}\;\mathrm{start}\;\mathrm{to}\;\mathrm{unstable}\;\mathrm{propagation}\\ K_{R}<K_{IC}^{P}\;\mathrm{stage}\;\mathrm{of}\;\mathrm{unstable}\;\mathrm{propagation} \end{array}\right.$$

It can be concluded that the double-K fracture criterion and Griffith’s criterion are still applicable in the analysis of the crack stability of the specimens in salt freeze–thaw cycles environment.

### 3.3. Choosing Critical Fracture Toughness as Freeze Thaw Damage Factor

#### Feasibility of Choosing $K_{IC}^{S}$ as Damage Factor

The RDME, $f_{cu.k}$, and $\Delta W_{n}$ are frequently used as damage factors. However, some deeper mechanisms need to be clarified, in that $f_{cu.k}$ is not sensitive to the internal cracks of concrete. Furthermore, because the size of the specimen is large, and the specimen is completely immersed in the solution for freeze–thaw, the same dryness of the specimen surface cannot be guaranteed during the mass weighing, and the spalling part of the specimen cannot be accurately taken out for weighing. Therefore, there will be a large error in measuring the spalling amount, which will affect the test results. To summarize, $f_{cu.k}$ and $\Delta W_{n}$ are not preferred for damage measurement, and the RDME is more familiar.

SEM was used to observe the surface of concrete specimens after salt freeze–thaw by researchers [11] (Lei et al. 2015). The results show that the essence of damage caused by salt freeze–thaw is the formation and deepening of discontinuous micro surface. In that case, it is more reasonable to use the critical fracture toughness to measure the damage degree of concrete structure. The purpose of studying the fracture performance of concrete is to evaluate the crack resistance of concrete. The generation and expansion of concrete cracks is a process of discontinuous micro surface intersection and connection, and finally forming macro cracks. Therefore, using the critical fracture toughness as a damage factor is a direct way to reveal the extent of damage.

Some scholars took the fracture energy as the damage factor. However, fracture energy refers to the overall energy dissipated by unit area of concrete during the entire process of crack initiation, propagation, convergence, and eventual fracture under tensile stress. That means that fracture energy, like $\Delta W_{n}$ and RDME, measures the overall damage degree of concrete, so there is a good linear correlation between them. However, they underestimate the effect of local crack development on structural safety.

**Figure 9:**
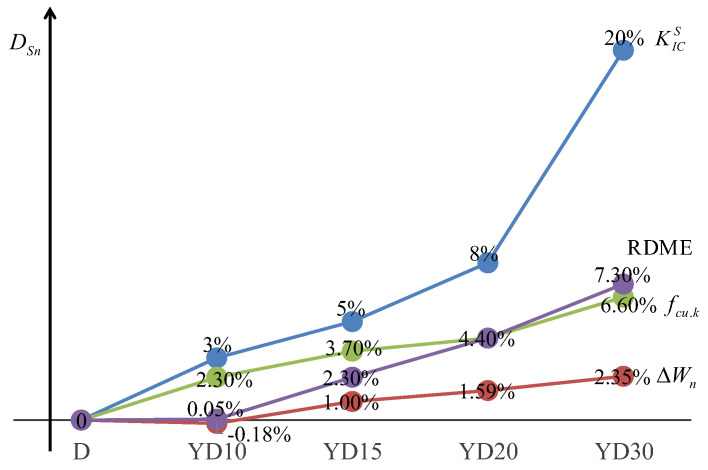
Damage factors in different cycles.

Consequently, a logical comparison was proposed after the normalization of several damage factors $D_{Sn}$ (mass, elastic model, compressive strength, and critical fracture toughness), as shown in Figure 9. The RDME and $f_{cu.k}$ are commonly used in damage assessment, where $K_{IC}^{S}$ is rarely used. However, the damage of specimens in salt freeze–thaw is currently underestimated (RDME 7.3%; $f_{cu.k}$ 6.6%; $K_{IC}^{S}$ 20%), for the concrete is often damaged by crack propagation before reaching its ultimate load. Using the existing damage assessment criteria will ignore the potential safety hazards. In that case, using $K_{IC}^{S}$ as damage factors is more reasonable and higher security.

Damage factors $D_{Sn}$ was calculated by Equation (12).

(12) $$D_{Sn}=S_{n}/S_{0}$$

where $S_{n}$ is the damage factor in n cycles, and $S_{0}$ is the initial damage factor.

Our proposed damage model is more robust and accurate for predicting damage degree in concrete facing Na-Cl&FTC than the existing model. It can be concluded that the trend of specimens weight loss reveal a close connection between weight loss and fracture properties.

## 4. Conclusions

In this paper, the fracture behavior of concrete attack by Na-Cl&FTC was mainly studied. Based on the results of this experimental work, the following conclusions are drawn:

(1) Compressive strength and RDME both decrease with increasing FTC times; specimens in the Na-Cl solution have lower compressive strength and FTC resistance compared with specimens in pure water. The reduction relationship of concrete fracture behavior between freeze–thaw damage in Na-Cl solution and pure water were three times.
(2) The feasibility of double-K fracture model was confirmed, $F_{\max}$, $G_{f}$, $F_{Q}$, $K_{IC}^{S}$ are all decreasing with increasing FTC times and demonstrate that freeze–thaw damage reduces concrete’s toughness or fracture behavior, which are sensitive to freeze–thaw cycles in salt solution; there is good correlation both between fracture energy and freeze–thaw cycles and between the loss of relative dynamic elastic modulus and the loss of fracture energy, which can be used to evaluate fracture behavior subjected to freeze–thaw damage; other parameters like $V_{Q}$, $V_{C}$, $K_{IC}^{Q}$ are insensitive and more cycles were needed.
(3) The CEB-FIP Model Code 1990 was used to calculated the strain-softening curves. This model is more accurate than Petersson and Reinhardt. The value ${\left(K_{IC}^{C,e}-K_{IC}^{C,t}\right)}^{2}$ calculated using CEB-FIP Model Code 1990 is smaller than Petersson and Reinhardt. The stability of crack propagation was studied.
(4) The damage of specimens in salt freeze–thaw is currently underestimated, and using $K_{IC}^{S}$ as damage factors is more reasonable and provides higher accuracy than $G_{f}$, RDME, $f_{cu.k}$, and $\Delta W_{n}$.

## Figures and Tables

**Table 1:** Concrete mix proportion (by weight) and $f_{cu.k}$ at 28 days.

Strength	Cement	Sand	Aggregate	Water	Superplasticizer	Compressive Strength
(MPa)	(kg/m^3^)	(kg/m^3^)	(kg/m^3^)	(kg/m^3^)	(kg/m^3^)	(150 mm Cube, MPa)
C50	481.48	635.93	1283.7	192.59	4.44	50.3

**Table 2:** Chemical composition of raw materials (wt.%).

Mineral Admixture	CaO	SiO_2_	Al_2_O_3_	Fe_2_O_3_	SO_3_	MgO	Loss
OPC	59.6	22.1	6.0	4.2	3.7	2.5	2.53

**Table 3:** Details of specimens.

Types of Specimens	Specimens	Number of Specimens	Freeze–Thaw Cycles	Solution
SENB/cube	D	4	0	None
	SD30	4	30	Water
	YD10	4	10	Na-Cl
	YD15	4	15	Na-Cl
	YD20	4	20	Na-Cl
	YD30	4	30	Na-Cl

**Table 4:** Weight loss of these TPB specimens (YD30/SD30).

Freeze–Thaw Cycles		YD30				SD30			
		1	2	3	4	1	2	3	4
0	$W_{0}$ (kg)	1.701	1.731	1.711	1.661	1.689	1.702	1.714	1.738
5	$W_{n}$ (kg)	1.703	1.733	1.713	1.663	1.691	1.704	1.716	1.740
	$\Delta W_{n}$	−0.118%	−0.116%	−0.117%	−0.120%	−0.118%	−0.118%	−0.117%	−0.115%
10	$W_{n}$ (kg)	1.704	1.734	1.714	1.664	1.692	1.705	1.717	1.741
	$\Delta W_{n}$	−0.176%	−0.173%	−0.175%	−0.181%	−0.178%	−0.176%	−0.175%	−0.173%
15	$W_{n}$ (kg)	1.684	1.712	1.695	1.649	1.668	1.678	1.696	1.724
	$\Delta W_{n}$	0.999%	1.098%	0.935%	0.722%	1.243%	1.410%	1.050%	0.806%
20	$W_{n}$ (kg)	1.674	1.701	1.689	1.635	1.659	1.671	1.684	1.712
	$\Delta W_{n}$	1.587%	1.733%	1.286%	1.565%	1.776%	1.821%	1.750%	1.496%
25	$W_{n}$ (kg)	1.666	1.694	1.679	1.627	1.653	1.664	1.676	1.703
	$\Delta W_{n}$	2.058%	2.137%	1.870%	2.047%	2.131%	2.233%	2.217%	2.014%
30	$W_{n}$ (kg)	1.661	1.689	1.671	1.622	1.651	1.659	1.671	1.695
	$\Delta W_{n}$	2.352%	2.426%	2.338%	2.348%	2.250%	2.526%	2.509%	2.474%

**Table 5:** Compressive strength ($f_{cu.k}$) and RDME of these cube specimens.

$f_{cu.k}$	FTC Times					
	D	SD30	YD10	YD15	YD20	YD30
Specimen-1	49.44	48.51	48.41	47.95	47.84	46.60
Specimen-2	50.22	49.17	48.77	47.87	47.40	47.48
Specimen-3	50.89	50.02	49.81	49.01	48.97	46.20
Specimen-4	49.79	48.79	48.52	47.65	47.15	45.77
Loss of $f_{cu.k}$	0%	1.9%	2.3%	3.7%	4.4%	6.6%
Loss of RDME	0%	1.8%	0.05%	2.3%	4.4%	7.3%

**Table 6:** Fracture parameters of these specimens.

No.	$\begin{array}{c} F_{Q}\\ \left(\mathrm{kN}\right) \end{array}$	$\begin{array}{c} \;F_{\max}\\ \left(\mathrm{kN}\right) \end{array}$	$\begin{array}{c} V_{C}\\ \mu m \end{array}$	$\begin{array}{c} K_{IC}^{Q}\;\;K_{IC}^{S}\\ \;\mathrm{MPa}\cdot m^{0.5} \end{array}$		$\begin{array}{c} a_{c}\\ m \end{array}$	$\begin{array}{c} G_{f}\\ N/\mathrm{mm} \end{array}$	$\frac{F_{Q}}{F_{\max}}$	$\frac{K_{IC}^{Q}}{K_{IC}^{S}}$
D-1	0.92	1.322	23.24	0.501	1.139	0.0234	0.124	69.59%	0.440
D-2	0.96	1.344	23.20	0.523	0.971	0.0203	0.145	71.43%	0.539
D-3	0.89	1.310	23.10	0.483	1.097	0.0229	0.113	67.65%	0.440
D-4	0.89	1.342	23.20	0.485	1.107	0.0226	0.117	66.32%	0.438
AVG	0.92	1.330	23.19	0.498	1.078	0.0223	0.125	69.00%	0.464
YD10-1	0.88	1.130	25.32	0.463	1.061	0.0248	0.117	75.27%	0.436
YD10-2	0.72	1.230	25.07	0.479	1.103	0.0240	0.129	71.54%	0.435
YD10-3	0.74	1.135	24.25	0.403	1.066	0.0234	0.123	59.92%	0.378
YD10-4	0.81	1.130	22.48	0.441	0.939	0.0228	0.119	71.68%	0.470
AVG	0.79	1.156	24.28	0.447	1.042	0.0238	0.122	69.6%	0.430
YD15-1	0.62	1.300	28.55	0.338	1.121	0.0234	0.104	47.71%	0.301
YD15-2	0.54	0.991	30.76	0.294	0.856	0.0234	0.120	54.50%	0.343
YD15-3	0.68	1.134	29.16	0.300	0.947	0.0271	0.116	59.99%	0.299
YD15-4	0.77	1.300	29.51	0.419	1.161	0.0240	0.108	59.25%	0.361
AVG	0.65	1.181	29.50	0.338	1.021	0.0245	0.112	55.00%	0.326
YD20-1	0.61	1.125	28.86	0.349	0.807	0.0202	0.086	56.90%	0.432
YD20-2	0.64	1.172	26.41	0.349	1.094	0.0247	0.104	54.63%	0.319
YD20-3	0.61	1.153	29.33	0.332	1.007	0.0236	0.112	52.89%	0.331
YD20-4	0.65	1.193	27.33	0.354	1.039	0.0236	0.110	54.47%	0.341
AVG	0.63	1.161	27.98	0.346	0.987	0.0230	0.103	55.00%	0.356
YD30-1	0.68	1.332	28.74	0.370	1.049	0.0219	0.080	51.05%	0.353
YD30-2	0.53	0.852	22.25	0.289	0.701	0.0226	0.077	62.24%	0.412
YD30-3	0.61	1.023	25.33	0.332	0.847	0.0227	0.092	59.63%	0.393
YD30-4	0.65	1.132	27.17	0.354	0.860	0.0212	0.079	57.42%	0.412
AVG	0.62	1.085	25.87	0.336	0.864	0.0221	0.082	58.00%	0.393
SD30-1	0.43	0.942	26.25	0.234	0.878	0.0234	0.121	45.67%	0.267
SD30-2	0.64	1.282	28.12	0.349	1.161	0.0203	0.126	49.92%	0.300
SD30-3	0.61	1.169	27.74	0.332	1.128	0.0229	0.112	52.18%	0.295
SD30-4	0.59	1.084	26.11	0.322	1.052	0.0241	0.119	54.43%	0.306
AVG	0.57	1.119	27.06	0.309	1.055	0.0223	0.119	51.00%	0.292

**Table 7:** Fracture toughness of these cube specimens.

	D	SD30	YD10	YD15	YD20	YD30
$K_{IC}^{Q}$	0.309	0.498	0.447	0.338	0.346	0.336
$K_{IC}^{S}$	1.055	1.078	0.884	1.021	0.987	0.864
$K_{IC}^{C,e}$	0.746	0.58	0. 437	0.683	0.641	0.528
$K_{IC}^{C,t}$ by Petersson	0.7309	0.5689	0.4195	0.6640	0.5809	0.5141
$K_{IC}^{C,t}$ by CEB-FIP	0.741	0.5713	0.4218	0.6752	0.5965	0.5266
$K_{IC}^{C,t}$ by Reinhardt	0.7261	0.5639	0.4164	0.6677	0.5811	0.5639
${\left(K_{IC}^{C,e}-K_{IC}^{C,t}\right)}^{2}$	0.000025	0.000076	0.000231	0.000061	0.00198	0.000002

## Data Availability

Not applicable.

## List of Symbols

$G_{f}$ Fracture Energy (N/mm)	RDME Relative Dynamic Modulus of Elasticity (GPa)
$f_{cu.k}$ Compressive Strength (MPa)	$\Delta W_{n}$ Weight Loss of Specimens at every 5 Cycles (%)
$W_{0}$ Average Weight of Concrete Specimens before Freeze–Thaw Cycles (kg)	
$W_{n}$ Average Weight of Concrete Specimens at every 5 Cycles in Water and NaCl Solution (kg)	
$c_{i}$ Flexibility Coefficient (µm/kN)	E Calculated Elastic Modulus (GPa)
t Thickness of the Specimens and t = 0.05 m	DME Dynamic Modulus of Elasticity (GPa)
$a_{0}$ Initial Notch Depth of the Specimen and $a_{0}$ = 0.015 m	
$h_{0}$ Initial Crack Width and $h_{0}$ = 0.003 m	h Height of the Specimens and h = 0.05 m
$a_{c}$ Critical Notch Depth of the Specimen (m)	$F_{\max}$ Maximum Load of the Specimen (kN)
$V_{c}$ Critical Crack Mouth Opening Displacement (µm)	$F_{Q}$ Initial Cracking Load (kN)
$K_{IC}^{Q}$ Initial Fracture Toughness (MPa·m^0.5^)	m Weight of the Specimen (kN)
g Acceleration of Gravity and g = 9.8 m/s^2^	S Span of the Specimen and S = 0.2 m
$K_{IC}^{S}$ Critical Fracture Toughness (MPa·m^0.5^)	$A_{lig}$ Fracture Ligament Area
$w_{0}$ Area between F and the Opening Displacement Curve	$f_{t}$ Tensile Strength (MPa)
$V_{Tc}$ Critical Crack Tip Opening Displacement (µm) and $a_{w_{0}}$ Corresponding Notch Depth	
